# Advances in immune checkpoint inhibitors induced-cardiotoxicity

**DOI:** 10.3389/fimmu.2023.1130438

**Published:** 2023-02-23

**Authors:** Xiang Li, Wenying Peng, Jiao Wu, Sai-Ching Jim Yeung, Runxiang Yang

**Affiliations:** ^1^ Department of the Second Medical Oncology, The Third Affiliated Hospital of Kunming Medical University, Kunming, Yunnan, China; ^2^ Department of Emergency Medicine, The University of Texas MD Anderson Cancer Center, Houston, Texas, TX, United States

**Keywords:** cardiotoxicity, immune checkpoint inhibitors, immune-related adverse events, Myocarditis, Pericarditis, Vasculitis

## Abstract

Immune checkpoint inhibitors (ICIs) are approved as the first-line drug for treating many cancers and has shown significant survival benefits; however, it also causes immune-related adverse events (irAEs) while activating the immune system, involving multiple organs. Among them, cardiovascular immune-related adverse events (CV-irAE) are rare, but common causes of death in ICIs treated cancer patients, which manifest as myocardial, pericardial, vascular and other cardiovascular toxicities. Therefore, it is important that irAEs, especially CV-irAE should be carefully recognized and monitored during the whole ICIs treatment because early detection and treatment of CV-irAE can significantly reduce the mortality of such patients. Consequently, it is urgent to fully understand the mechanism and management strategies of CV-irAE. The effects of ICIs are multifaceted and the exact mechanism of CV-irAE is still elusive. Generally, T cells identify tumor cell antigens as well as antigen in cardiomyocytes that are the same as or homologous to those on tumor cells, thus causing myocardial damage. In addition, ICIs promote formation of cardiac troponin I (cTnI) that induces cardiac dysfunction and myocardial dilatation; moreover, ICIs also increase the production of cytokines, which promote infiltration of inflammation-linked molecules into off-target tissues. Currently, the management and treatment of cardiovascular toxicity are largely dependent on glucocorticoids, more strategies for prevention and treatment of CV-irAE, such as predictive markers are being explored. This review discusses risk factors, potential pathophysiological mechanisms, clinical manifestations, and management and treatment of CV-irAE, guiding the development of more effective prevention, treatment and management strategies in the future.

## 1 Introduction

During tumorigenesis, tumor cells inhibit the activation and effector process of T cells by hijacking immune checkpoints molecules, then evade the surveillance and attack of the immune system. Thus, immune checkpoint related to the regulation of T-cell activity is an important target for anti-tumor therapy (1). Tumor microenvironmental factors also modify the anti-tumor immune response, such as T-cell infiltration and expression of immune checkpoint proteins (2). Currently, the main immune checkpoints include cytotoxic T-lymphocyte antigen 4 (CTLA-4), programmed cell death receptor 1 (PD-1), programmed cell death ligand 1 (PD-L1) and lymphocyte activation gene 3 protein (LAG3). Immune checkpoint inhibitors (ICIs) are now approved for treating many malignancies and significantly prolonged the survival of cancer patients (3–6). At the beginning of ICIs application, reports of immune-related adverse events (irAEs) were rare and did not attract broad attention. However, with the rapidly increased use of ICIs and the improvement of patients’ survival, the importance of cardiovascular immune-related adverse events (CV-irAE) therapy has come to the forefront. Despite its low incidence, immune-related adverse events (CV-irAEs) require high attention from clinicians (7). Therefore, through exploring the underlying mechanisms of CV-irAE, we developed more effective prevention, treatment, and management strategies, thus improving the quality of life and patients’ survival. Herein, we review the pharmacological mechanisms of ICIs, current research progression in CV-irAEs epidemiology, risk factors, potential pathophysiological mechanisms as well as clinical manifestation, the management and treatment of CV-irAEs mentioned in guidelines and literatures. The above statements are gross generalizations based on our synthesis of the current litereature.Some statements are not accepted by all, but most of them are based on guidelines published by prestigious professional organizations.

## 2 Epidemiology

Current reports about epidemiology of CV-irAE are limited because of its low incidence (8). CV-irAEs occur as early as a few days after ICIs initiation, but may also present late until one year after ICIs treatment, the median onset time of CV-irAE was 34 days after starting ICIs (9, 10). In a Danish national study, patients with lung cancer and malignant melanoma had a higher risk rate of CV-irAE in patients treated with ICIs than those who did not receive ICIs therapy (11). Wang et al. (12) performed a retrospective analysis of published irAEs queried in the pharmacovigilance database (Vigilyze) and found that myocarditis had the highest fatality rate among all CV-irAEs (39.7%). Rubio et al. analyzed 1265 papers published before August 31, 2020 and found the total incidence of CV-irAE was about 1.3%, among them myocarditis was the most common irAE, accounting for 50.8%. Notably, a high mortality rate of 24.6% of patients died due to CV-irAE (13). In this study, ICIs included ipilimumab, tremelimumab, nivolumab, pembrolizumab, atezolizumab, durvalumab and avelumab. In addition to these ICIs, there are emerging ICIs, which may also occur CV-irAEs such as relatlimab, a emerging monoclonal antibody that targets LAG-3, relatlimab had a higher incidence in myocarditis (14, 15). Since relatlimab has been approved soon, relatlimab related cardiotoxicity needs to be further explored. The incidence of CV-irAE appears to increase in recent years, probably due to the increased scope and frequency of use of ICIs and the heightened awareness of cardiotoxicity (16–18). However, the real-world prevalence of CV-irAE may be higher than expected, and we currently lack the support of large-sample clinical studies that could offer further in-depth investigation (9, 17).

## 3 Risk factors for CV-irAE

The risk factors of CV-irAE need further investigation, dual ICIs combination therapy is the greatest risk factor for CV-irAE over other risk factors such as autoimmune diseases (19). Several investigations have also confirmed that dual ICI leads to a higher incidence of CV-irAE than monotherapy or ICI plus chemotherapy (19, 20). A meta-analysis of CV-irAE concluded that the incidence was 3.1% for ICI monotherapy, 2.5% for ICI plus chemotherapy and 5.8% for dual ICIs treatment (anti-PD-1 plus anti-CTLA-4/anti-PD-1 plus anti-PD-L1) (13). The emerging bispecific antibody also causes CV-irAE. The incidence of CV-irAE is 0.9% in 458 patients treated with Cadonilimab (anti-PD-1/CTLA-4) (21). Cardiotoxicity of AK112 (NCT04047290)—anti-PD-1/VEGF and IBI318(NCT03875157)—anti-PD-1/PD-L1 has not been reported.

It was demonstrated that the PD-1 modulates radiation-induced cardiotoxicity in an animal model, acute toxicity was increased with anti-PD-1 treatment in mice with radiotherapy, but further research is needed to get a deep insight (22). Osaka Medical School in Japan established a mouse model of experimental autoimmune myocarditis (EAM) by administration of PD-1 antibodies in mice (23). The study indicated that ICIs-induced autoimmune myocarditis may be related to autoimmunity prior to ICIs administration (23). CV-irAE is more frequently reported in patients diagnosed with autoimmune diseases (24). In a retrospective case-match control study comparing 251 ICI-treated patients who had autoimmune diseases with 251 ICI-treated patients who did not have autoimmune diseases, the risk of CV-irAEs was higher in patients with autoimmune diseases than those without (hazard ratio:1.77) (25).

In addition, the observation of sporadic ICIs-associated myocarditis cases revealed that patients with diabetes were more common in these cases (9, 26). In addition, the patients’ pre-existing cardiovascular risk factors (age ≥80 years, hypertension, diabetes mellitus and chronic kidney disease) and the presence of cardiovascular toxicity caused by previous anti-neoplastic drugs should also be brought to our attention (27). Comparing 35 patients who had ICIs-related myocarditis with 105 ICIs-treated patients who did not have ICIs-related myocarditis, 34% of patients with ICIs-related myocarditis had pre-existing diabetes but only 13% of ICIs-treated patients without myocarditis had diabetes (28).

## 4 Mechanism of CV-irAE

### 4.1 Pharmacological mechanism of ICIs

**Figure 1:**
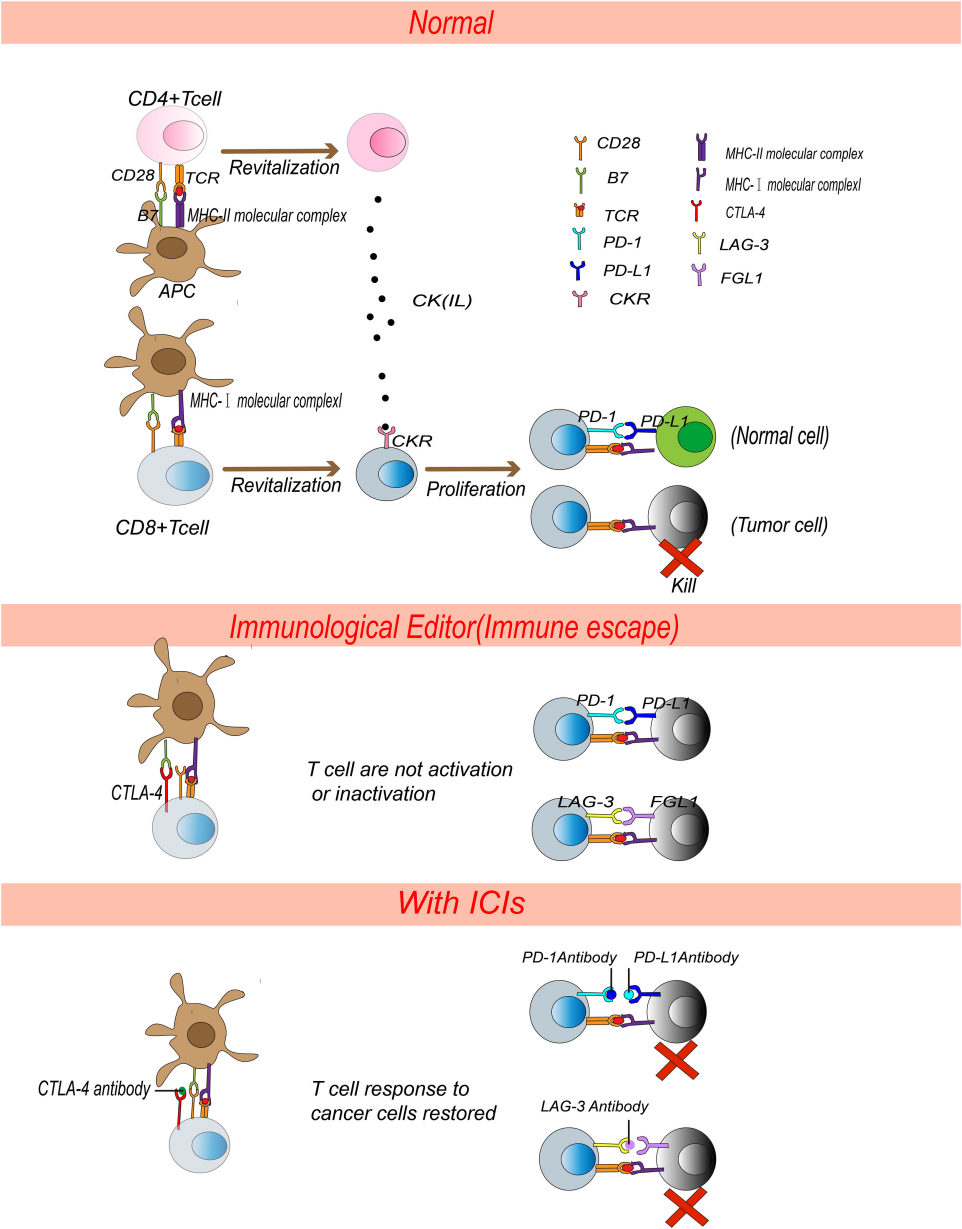
Pharmacological mechanism of ICIs. APCs present MHC molecular complexes to TCR on T cells and activate T cells. CD4+ T cells secrete cytokines and stimulate CD8+ T cells proliferation. Activated CD8+ T cells kill tumor cells precisely. Normally, PD-L1 binds to PD-1, FGL-1 binds to LAG-3, inactivating CD8+ T cells and leading to autoimmune tolerance. After immunoediting, tumor cells express PD-L1 and FGL-1 and T cells express CTLA-4 and LAG-3, receptors on T cells bound with ligands on tumor cells or APCs, which will inactivate T cells. ICIs devitalized the PD-1/PD-L1, LAG-3/FGL-1 and CTLA-4/B7 signals and reactivated T cells to kill tumor cells.

The immune system plays an important role in the surveillance and wiping malignant cells. T cells undergo positive and negative selection in thymic to ensure self-tolerance and specific recognition of abnormal cells (including cancer cells) (29). Tumor cells presenting or releasing tumor antigens are engulfed by antigen presenting cells (APCs), which process tumor antigens and present MHC-I and MHC-II molecular complexes to CD8+ T-cell and CD4+ T-cell receptors then accurately identify cancer cells. A combination of B7, on the surface of APCs, and CD28, on the surface of T cells, constitute synergistic signals in T cells activation, the combination of CD28-B7 lead to cytoskeleton remodelling, cytokines secretion and T cells differentiation. Activated CD4+ T cells secrete cytokines to stimulate CD8+ T cells proliferation in lymph nodes. Activated CD8+ T cells can reach the tumor through circulation, recognize the MHC-I molecular complex on the tumor cells, and kill tumor cells (30–33). Activated CTLA-4, PD-1 and LAG-3 to protect the host from self-attack by abnormally activated T cells (6, 34, 35). CTLA-4, a CD28 homolog, has stronger affinity than CD28, and can induce trans-endocytosis of B7 ligands to reduce the co-stimulatory signal (36–38). PD-1, combined with PD-L1, negatively mediates T cell proliferation and activation (39, 40). CTLA-4 not only competes with CD28 for B7 but also induces regulatory T cells (Treg, inhibitory immune cells) to death, leading to unbalance between Treg and cytotoxic T cells (41, 42). CTLA-4 monoclonal antibody clears Treg in tumor effectively through FcR mediated ADCC (antibody-dependent cell-mediated cytotoxicity), thus relieving immunosuppressive of Treg to achieve anti-tumor (42–44). However, Treg cells are important in peripheral tolerance (45). Reducing peripheral Treg cells lead to the immune system attacking organism, resulting in serious side effects (45, 46). PD-1 plays an important role in T-cell homeostasis and inflammatory inhibition in peripheral tissues (34, 47). Lymphocyte activation gene 3 protein (LAG3) is a negative immunomodulator that regulates the function of T cells and dendritic cells (DC) by binding with MHC-II (6). LAG-3 has an intracellular short tail domain that inhibits the function of LAG-3 in effector CD4+ T cells and an extracellular domain similar to CD4 but possess higher affinity to combine with MHC-II than CD4 (6, 48). FGL1, the ligand of LAG-3, expressed on the surface of cancer cells. When FGL1 combines with LAG-3 on the surface of T cells, immune system mistake cancer cells as normal, contributing to immune-escape of tumor cells (49). After immunoediting (50–52), tumor cells would also express immune checkpoint, so ICIs are designed to reactivate anti-tumor immune response by targeting specific immune checkpoint (**Figure 1**). Therefore, CTLA-4, PD-1, PD-L1and LAG3 inhibitors have been approved for clinical treatment in several cancer types by Food and Drug Administration (FDA) (1, 53). In addition, new-type ICIs through targeting inhibitory receptors [e.g,. T cell immunoglobulin domain and mucin domain-3 (TIM-3), T cell Ig and ITIM domain (TIGIT) and BTLA (CD272)] and ligand of the B7 family [e.g., V-domain Ig suppressor of T cell activation (VISTA), B7-H3] are being actively investigating and developed for clinical trials in increasing numbers (54–57).

### 4.2 Potential pathophysiological mechanisms of CV-irAEs

**Figure 2:**
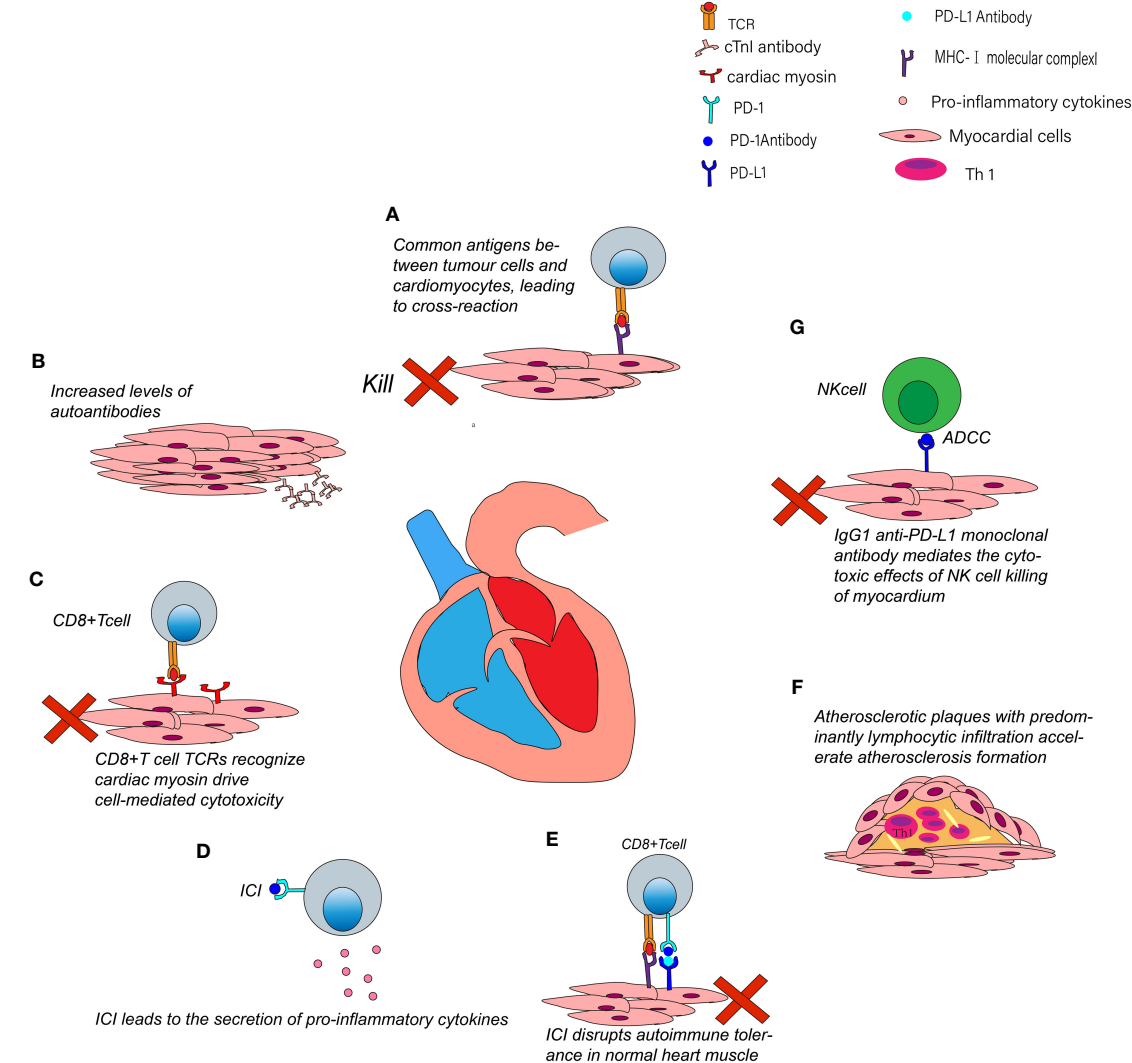
Possible mechanisms of CV-irAE. **(A)** Activated T cells not only attack tumor cells but also cross-reactivate with cardiac muscle. **(B)** Cardiac myocytes secrete cTnI antibodies after using ICIs. **(C)** Myosin-specific T cells TCRs can recognize myosin and drive cytotoxic T-cell-mediated killing. **(D)** ICIs can lead to increased levels of pro-inflammatory cytokines. **(E)** Systemic application of ICI may disrupt immune homeostasis between cytotoxic T cells and Tregs. **(F)** ICIs may contribute to plaques progression and coronary events. **(G)** Anti-PD-L1 monoclonal antibodies may mediate NK cells killing cardiomyocytes through the ADCC pathway.

The mechanism of CV-irAE might be ICIs disrupt the autoimmune tolerance of myocardial cell (58). irAEs are reversible in most cases treated appropriately; however, heart is a vital organ so CV-irAE can be fatal (20, 59). Though the effects of ICIs are multifaceted, the exact mechanisms of CV-irAE are still elusive (52) (**Figure 2**).

#### 4.2.1 The common antigens in tumor cells and cardiomyocytes leading to cross-reaction

T cells identify tumor cell antigens as well as antigen in cardiomyocytes same with or homologous to those on tumor cells simultaneously. In two cases of fulminant myocarditis caused by ICIs, postmortem found that T cell marker (CD3) was positive in myocardial and skeletal muscle infiltrating cells. T cells receptor sequence revealed that patients had high frequency of shared T cell receptor sequences in cardiac and skeletal muscle and tumor infiltrating cells (20). Taken together, these suggest that activated T cells not only attacked tumor cells but also caused cross-reaction with common antigens on skeletal and cardiac muscles, but the specific antigen was not identified in the study. T cells-mediated immune responses in the heart may cause abnormal heart electrical rhythm and irreparable damage to myocardium (58).

#### 4.2.2 Increase of autoantibody

ICIs promote the formation of autoantibodies. Lack of PD-1 caused autoimmune dilated cardiomyopathy in mouse model with *Pdcd1* gene knockout, and high titers of circulating immunoglobulins (IgGs) deposited on surface of mouse cardiomyocytes (60). Subsequent experiments showed that the autoantibodies are against cTnI. cTnI induced cardiac dysfunction and myocardial dilatation by means of chronically stimulating influx of calcium ions in cardiomyocytes (61).

#### 4.2.3 Cardiac myosin drive cell-mediated cytotoxicity

*Won et al.* (62) used anti-PD-1 monoclonal antibodies to induce the development of myocarditis in mice and they found that myosin-specific T cells were increased in such mice. *Axelrod et al. (* 63) has established P*dcd1^-/-^/Ctla4* ^+/-^ mouse model to characterize ICIs-related myocarditis. Single-cell RNA and T cell receptor (TCR) sequencing were arranged and found increasing CD8+T cells in ICIs-related myocarditis. They subsequently found that specific TCRs recognize α-myosin, suggesting α-myosin may drive cytotoxic T-cell-mediated killing.

#### 4.2.4 High level of cytokines

Cytokines that recruit immune cells to tumor microenvironment are significant modulators for immune response (58). ICIs lead to increased pro-inflammatory cytokines, which activate T-cells proliferation and result in anti-tumor immune response (64–66). *Tarhini* et al. (64) found that restraining immune checkpoints result in higher circulating pro-inflammatory cytokines [interferon (IFN)-γ, tumor necrosis factor (TNF)-α, interleukin (IL), and granulocyte macrophage colony-stimulating factor (GM-CSF)]. Those cytokines contribute to ICIs penetration into non-target organs (including cardiovascular cells) (64, 65, 67, 68).

#### 4.2.5 Immune tolerance

Immune checkpoints inhibit T cells activation is called immune tolerance. For example, the PD-1/PD-L1 pathway prevents T cells overactivation to maintain immune balance. Blocking PD1/PD-L1will not only promote anti-tumor immunity but also inhibit Treg cells and Forkhead Box P3 (FOXP3) expression, leading to loss of self-tolerance (69). Treg cells have an effective role in keeping peripheral tolerance. Systemic application of ICIs may disrupt immune homeostasis between cytotoxic T cells and Treg cells in normal myocardial tissue, causing the development of cardiotoxicity (70, 71).

#### 4.2.6 Atherosclerosis

Atherosclerosis is the inflammation of large arteries (72). PD-1 and CTLA-4 restrain formation of atherosclerosis. PD-1 deficient bone marrow progenitor cells up-regulate genes involved in cholesterol synthesis and ingestion, leading to elevated cholesterol (73). Blockading CTLA-4 increases T cells abundance in plaques and exacerbates atherosclerosis in mouse model (74). *Banerjee et al.* (75) found that senescence-associated secretory phenotype (SASP) are intersections of cancer and cardiovascular events, and SASP can aggravate atherosclerosis. More importantly, ICIs can lead to therapy-induced SASP and accelerate atherosclerosis, so atherosclerosis should be monitored while using ICIs ( (75, 76). A matched cohort study (77) showed that patients treated with ICIs have a 3-fold increase risk for cardiovascular events (77). Autopsies were performed on tumor decedents who received ICIs and those who did not, and the result showed that the ratio of CD3/CD68 was significantly elevated in atherosclerotic plaques among patients undergoing ICIs (78). After treated with ICIs, inflammation in atherosclerotic plaques was dominated by lymphocytes rather than macrophages, which is usually primary cell of atherosclerosis (78). Lymphocytes have a significant effect on the development of atherosclerosis, and in mouse model Th1 cells promote the development of atherosclerosis by secreting IFN-γ (79–81). In summary, this evidence suggests that ICIs may contribute to plaques and coronary events by altering the type of inflammation in atherosclerotic plaques (78).

#### 4.2.7 ADCC(antibody-dependent cell-mediated cytotoxicity)

ICIs interact with proteins expressed on myocardial tissue, such as CTLA-4, FGL1, LAG-3, PD-1 and PD-L1, resulting in complement-mediated tissue injury. The Fc region of human IgG1 monoclonal antibodies binds to receptors on natural killer (NK) cells mediating ADCC. Therefore, most immune checkpoint monoclonal antibodies are IgG4 that do not mediate ADCC; however, avelumab is a human IgG1 anti-PD-L1 monoclonal antibody. Theoretically, the antibodies, bind to PD-L1 on surface of cardiomyocytes, and may mediate killing of cardiomyocytes by NK cells through ADCC (82–84).

## 5 Clinical manifestations of CV-irAE

CV-irAE may appear as symptoms from the myocardial, pericardial and vascular system of the body (71, 85).

### 5.1 Myocardial disease

#### 5.1.1 Myocarditis

Myocarditis appears as early as 2 weeks after ICIs, and the median time is 65 days (86, 87). Myocarditis is the most frequent CV-irAE, possibly shown as asymptomatic myocarditis with an increase of cardiac biomarkers, or could be severe cardiac damage, even break out fulminant or life-threatening manifestations such as cardiogenic shock, heart failure, arrhythmias, advanced atrioventricular block or ventricular tachycardia (9, 20, 86, 88). Progression of ICIs-associated myocarditis is fulminant but can also be doubted by clinical symptoms, electrocardiography and biomarkers [troponin, brain natriuretic peptide (BNP)] and imaging (17). Myocardial biopsy is the definitive standard to identify myocarditis. The typical myocarditis clinical symptoms include palpitations, chest pain, heart failure and a range of other manifestations (89).

#### 5.1.2 Takotsubo syndrome

Takotsubo syndrome usually appears between 15 weeks to 8 months after ICIs; however, due to its low incidence, epidemiological data are lacking and the literatures are still limited to only case reports (87). Takotsubo syndrome is an acute and transient syndrome of regional left ventricular insufficiency (90). It was first identified in Japan and characterized by myocardium dilating like a balloon and may lead to several dangerous symptoms. It was usually caused by severe stress. For clinical examination, echocardiograph shows apical or mid-left ventricular dyskinesia and troponin and NT-proBNP will elevate (91–93). A melanoma patient present takotsubo syndrome after ICIs combination therapy, and echocardiograph showed apical motion with ballooning, electrocardiogram showed V2-V6 ST elevation 1-2 mm. Cardiac MRI showed that left ventricular ejection fraction (LVEF) and systolic function returned to normal after corticosteroid treatment (94).

#### 5.1.3 Dilated cardiomyopathy

Activated T cells result in an immune response in vessels and myocardium lead to development of dilated cardiomyopathy (95). Similarly, epidemiological data on ICIs-induced dilated cardiomyopathy is insufficient due to its low incidence. Nishimura et al. (60) found that PD-1 knockout mice developed severe dilated cardiomyopathy. Subsequently, they found that cTnI can induce cardiac dysfunction and myocardial dilatation in cardiomyocytes. Although the clinical manifestation of Takotsubo syndrome and dilated cardiomyopathy is similar, the echocardiogram of dilated cardiomyopathy does not have apical ballooning syndrome (96). There is a dilated cardiomyopathy patient after Nivolumab treatment. Echocardiography shows diffuse hypokinesis and 20% Left Ventricular Ejection Fractions (LVEF), and myocardial biopsy found inflammatory cells and interstitial fibrosis, which did not consistent with myocarditis (96).

### 5.2 Pericardium

ICIs related pericardium include pericarditis and pericardial effusion (97, 98). In a retrospective study, the median onset time was 40 days for pericardial effusion in 6.7% of patients treated with ICIs (99). However, it can also occur very late after the start of ICIs. In a case of advanced non-small cell lung cancer, after Nivolumab the patient developed pericardial thickening and effusion after 18 months (100). Pericarditis and pericardial effusion may be asymptomatic or mild and life-threatening symptoms may also occur when hemodynamic is unstable (101). Breathlessness is the predominant symptom and is followed by tachycardia and chest pain (102). At the time of diagnosis, the effusion should be distinguished between tumor progression related pericarditis and CV-irAE by TTF-1 immunohistochemical staining (101).

### 5.3 Vascular diseases

#### 5.3.1 Vasculitis

Vasculitis caused by self-immune disorder can occur in vessels of all sizes (103). The incidence of ICIs-associated vasculitis is lower than 1%, and there was no clear epidemiological data on the median time (104). In a retrospective analysis of 1215 patients treated with ICIs, cardiovascular events occurred in approximately 1% of patients, and the median time to event was 97 days after ICIs (105, 106). Currently, irAE about vasculitis are reported mainly about large vessel and neurological vasculitis (107). ICIs lead to the activation of T cells and NK cells and the secretion of pro-inflammatory cytokines, resulting in inflammation of the vessel wall, revascularization and even vascular occlusion (108, 109). CT or MR can diagnose vasculitis that is characterized by diffuse peripheral thickening of the vessel wall, enhanced wall thickness, or thrombosis (104). Daxini et al. (107) reviewed 20 case reports that met the criteria by searching multiple medical databases, and the results showed that the most common types of ICIs-related vasculitis were macrovasculitis, such as giant cell arteritis (GCA). GCA is an inflammation of blood vessels that occurs in people older than 50 years and primarily affects the great and middle arteries, especially the extracranial branches of the aorta and external carotid arteries (108). The manifestations of GCA are various based on the vessels, leading to blindness, stroke and aneurysms (110). GCA can develop into vascular occlusion, leading to tissue ischemia and should be considered in patients with lately reported headache, visual impairment, claudication of the jaw and polymyositis rheumatica (PMR) symptoms (110). Atherosclerosis is an inflammation of the large arteries, and the primary outcome of accelerated atherosclerosis after ICIs was the occurrence of cardiovascular events (defined as a combination of myocardial infarction, coronary revascularization, and ischemic stroke) (77). A previous study found that atherosclerotic plaque can be ameliorated by the concomitant use of corticosteroids and statins (77).

## 6 Management and treatment of CV-irAE

### 6.1 Screening of baseline cardiovascular disease and risk factor

Prior to ICIs, physicians need to assess the potential cardiotoxicity of ICIs and educate patients to report suspicious symptoms to medical personnel in time (27). According to the European Society of Cardiology recommendations, risk factors of baseline include pre-existing cardiovascular disease, elevated cardiac biomarkers, and previous cardiotoxic antineoplastic drugs history (27). Baseline assessment includes physical examination and auxiliary examination, such as an electrocardiogram (ECG), echocardiogram and cardiac troponin and natriuretic peptide etc. Individualized baseline monitoring improves the survival of patients. Patients with abnormal baseline examination results (ECG, cardiac biomarkers) require therapy under the guidance of an integrated oncology and cardiology team (111, 112).

### 6.2 Monitoring of toxicity

Toxicity monitoring is performed through the process of ICIs, especially in patients with prior cardiac injury. Physicians should assess the possibility of CV-irAE at each follow-up visit. Monitoring of toxicity includes electrocardiogram, echocardiograms, myocardial markers, troponin and NT-proBNP: (1) electrocardiogram is routinely performed before each cycle of treatment, (2) patients are advised to follow-up regularly for echocardiograms and myocardial markers every 2-4 cycles and 6/12 months after ending using ICIs (86, 111, 113), (3) As recommended by 2021 American Society of Clinical Oncology (ASCO) guideline, there is no clear recommendation on the frequency of troponin and NT-proBNP (114). But a literature recommended testing troponin and NT-proBNP at baseline and 2-4 cycles (28).Toxicity monitoring may detect abnormal biomarkers prior to symptoms of CV-irAE. When troponin is elevated, physicians should look out for potential triad myositis-myositis, muscle weakness, and myocarditis. For patients suspicious of myositis, not only creatine kinase (CK) but also lactate dehydrogenase (LDH) should be tested because cardiotoxicity, myositis and myalgia may happen in the same patient. Once the patient appears suspicious clinical symptoms, a cardiology specialist should immediately be consulted (16, 17, 108, 111).

### 6.3 Diagnosis of CV-irAE

Diagnosis of CV-irAE is a challenge because there are many manifestations of CV-irAEs (115). The clinical presentation is similar to viral myocarditis which may confuse the diagnosis. The evaluation should include telemetry monitoring, serum marker (e.g., cardiac markers, CK, LDH), electrocardiogram and cardiac magnetic resonance (CMR) (116). Myocardial and vascular biopsies are the standard for diagnosing CV-irAE. Finally, diagnosis of CV-irAE should be integrated by a multidisciplinary cardio-oncology team (117).

### 6.4 Management and treatment of CV-irAE

#### 6.4.1 Grade and management

Management and treatment of CV-irAE mainly depend on toxicity grading, based on the dose and dosage of given immunosuppressants. ASCO, National Comprehensive Cancer Network (NCCN) and Chinese Society of Clinical Oncology (CSCO) have classified CV-irAE in detail (**Table 1**).

**Table 1:** Grading, manifestation, and management of CV-irAE.

Grade	Manifestation	Management
G1	No cardiovascular symptoms, cardiac biomarkers (creatine kinase, troponin) or electrocardiogram abnormalities	(1) If cardiac markers are mildly abnormal and remain stable, continue ICIs (2) Otherwise, ICIs should be discontinued until the markers recover to normal.
G2	Mild or moderate symptoms of activity or fatigue, abnormalities in cardiac biomarkers and electrocardiograms	(1) Discontinue ICIs (2) Be hospitalized (3) Cardiology consultation (4) High-dose steroids such as methylprednisolone pulse dosing 1 g/d IV for 3-5 days (5) ICIs should be used cautiously even if relevant indicators recover to normal.
G3	Cardiovascular symptoms at rest or after mild activity, ULN<cardiac biomarkers ≤ 3ULN, significant changes of echocardiographic, but no hypotension.	(1) Terminate using ICIs (2) High-dose steroids such as methylprednisolone pulse dosing 1 g/d IV for 3-5 days (3) MDT (4) Advanced Life Support in ICU
G4	Moderate to severe decompensation, hemodynamic instability (hypotension), and cardiac biomarkers >3ULN.	

ULN, upper limit of normal; ICU, intensive Care unit; MDT, Multi-Disciplinary Treatment.

#### 6.4.2 Similarities and differences between guidelines

Although the incidence of CV-irAE is low, ESMO/ASCO/NCCN/CSCO guidelines all consider CV-irAE as a disease characterized by diverse manifestations, rapid progression and high mortality. However, different recommended doses for glucocorticoid were given. ASCO guidelines recommended methylprednisolone 1-2 mg/kg•d, NCCN guidelines recommended pulsed methylprednisolone 1 g/d, and ESMO/CSCO guidelines recommend 500 to 1000mg/d (114, 116, 118) (**Table 2**).

**Table 2:** Recommended doses of glucocorticoids in different guidelines.

Guideline	Grade	Dose of steroids
2022ESMO		Methylprednisolone 500-1000 mg/d, 3 days or until clinically stable
2021ASCO	G2-G4	Methylprednisolone 1-2 mg/kg•d, oral or IV depending on the symptoms
2022NCCN	G1-G4	Methylprednisolone 1g/d IV, 3–5 days
2021CSCO	G2	Methylprednisolone 1-2 mg/kg•d, 3–5 days
	G3-G4	Methylprednisolone 500-1000mg/d, 3–5 days

#### 6.4.3 Steroid refractory CV-irAE

Other immunosuppressive agents (e.g., gammaglobulin, anti-thymocyte globulin, infliximab and morte-macrolimus) can be added if glucocorticoid mono-treatment fails after 24 hours. However, it should be noted that high-dose infliximab is forbidden if the patients have moderate to serious heart failure. Pacemakers can be installed in patients with arrhythmias if necessary, and mechanical hemodynamic support should be given promptly in critical patients (111, 118–120). All guidelines’ recommendations are based on high levels of evidence and recommended high doses of glucocorticoids. The different doses of glucocorticoid in guidelines maybe due to differences in panel references and reference areas. NCCN/ASCO have published many clinical practice guidelines with high level of evidence which have been recognized and followed by clinicians worldwide. The CSCO guidelines include a large number of toxicity data from China, and is more suitable for Chinese.

#### 6.4.4 Re-challenge of ICIs

ASCO guidelines recommended to terminate the use of ICIs in all patients with CV-irAE, while NCCN/CSCO guidelines recommend patients with grade 1-2 cardiotoxicity restart ICIs after symptom remission.

## 7 Emerging predictive markers

When patients show symptoms of CV-irAE, myocardial damage already exists. In addition to conventional markers, more sensitive predictive markers are needed to prevent myocardial damage in advance. Few studies of toxicity prediction of myocarditis have been reported, but a promising toxicity prediction marker of CV-irAE need to be further explored. Drobni et al. (121) conducted a case-control study in patients with ICIs myocarditis or without CV-irAE after ICIs treatment, showing that significantly higher neutrophil/lymphocyte ratio (NLR) was found in patients with ICI related myocarditis (121). Another study compared echocardiographic global longitudinal strain (GLS) in patients with ICIs myocarditis or without CV-irAE after ICIs treatment. They found that GLS is lower in patients with ICI related myocarditis and suggested a poor prognosis (122). In summary, NLR and GLS are potential makers of immune-mediated myocarditis.

## 8 Discussion

CV-irAE is lethal, so we expect to detect abnormalities before irreversible myocardial damage happens; therefore, more sensitive and reliable makers are urgently needed (123–125). Although ICIs have been widely used in treating cancer and achieved good results, a series of adverse events may happen after the application of ICIs. Cardiovascular toxicities are rare but usually fatal when it occurs. Therefore, we should continually explore the mechanism of CV-irAE, summarizing the cases that have occurred, strengthening awareness of prevention and improving the management of CV-irAE, and introducing of a new surveillance strategy.

## Author contributions

RY conceived the idea, XL wrote the manuscript, WP and JW revised the content and grammar of the manuscript. RY and S-CY reviewed the structure and content of the manuscript in the revision. All authors provided critical feedback and analysis and manuscript, and contributed to the final manuscript.
